# Cognitive Impairment After Intracerebral Hemorrhage: A Systematic Review of Current Evidence and Knowledge Gaps

**DOI:** 10.3389/fneur.2021.716632

**Published:** 2021-08-27

**Authors:** Thomas Potter, Vasileios-Arsenios Lioutas, Mauricio Tano, Alan Pan, Jennifer Meeks, Daniel Woo, Sudha Seshadri, Magdy Selim, Farhaan Vahidy

**Affiliations:** ^1^Center for Outcomes Research, Houston Methodist Research Institute, Houston Methodist, Houston, TX, United States; ^2^Department of Neurology, Beth Israel Deaconess Medical Center, Boston, MA, United States; ^3^Department of Nuclear Engineering, Texas A&M University, College Station, TX, United States; ^4^Department of Neurology and Rehabilitation Medicine, University of Cincinnati College of Medicine, Cincinnati, OH, United States; ^5^Glenn Biggs Institute for Alzheimer's and Neurodegenerative Diseases, University of Texas Health Science Center at San Antonio, San Antonio, TX, United States; ^6^Neurological Institute, Houston Methodist, Houston, TX, United States

**Keywords:** intracerebral hemorrhage, cognitive impairment, dementia, outcome, stroke, cerebral small vessel disease

## Abstract

**Background:** Cognitive impairment (CI) is commonly observed after intracerebral hemorrhage (ICH). While a growing number of studies have explored this association, several evidence gaps persist. This review seeks to investigate the relationship between CI and ICH.

**Methods:** A two-stage systematic review of research articles, clinical trials, and case series was performed. Initial search used the keywords [“Intracerebral hemorrhage” OR “ICH”] AND [“Cognitive Impairment” OR “Dementia OR “Cognitive Decline”] within the PubMed (last accessed November 3rd, 2020) and ScienceDirect (last accessed October 27th, 2020) databases, without publication date limits. Articles that addressed CI and spontaneous ICH were accepted if CI was assessed after ICH. Articles were rejected if they did not independently address an adult human population or spontaneous ICH, didn't link CI to ICH, were an unrelated document type, or were not written in English. A secondary snowball literature search was performed using reviews identified by the initial search. The Agency for Healthcare research and Quality's assessment tool was used to evaluate bias within studies. Rates of CI and contributory factors were investigated.

**Results:** Search yielded 32 articles that collectively included 22,631 patients. Present evidence indicates a high rate of post-ICH CI (65–84%) in the acute phase (<4 weeks) which is relatively lower at 3 (17.3–40.2%) and 6 months (19–63.3%). Longer term follow-up (≥1 year) demonstrates a gradual increase in CI. Advanced age, female sex, and prior stroke were associated with higher rates of CI. Associations between post-ICH CI and cerebral microbleeds, superficial siderosis, and ICH volume also exist. Pre-ICH cognitive assessment was missing in 28% of included studies. The Mini Mental State Evaluation (44%) and Montreal Cognitive Assessment (16%) were the most common cognitive assessments, albeit with variable thresholds and definitions. Studies rarely (<10%) addressed racial and ethnic disparities.

**Discussion:** Current findings suggest a dynamic course of post-ICH cognitive impairment that may depend on genetic, sociodemographic and clinical factors. Methodological heterogeneity prevented meta-analysis, limiting results. There is a need for the methodologies and time points of post-ICH cognitive assessments to be harmonized across diverse clinical and demographic populations.

## Introduction

Intracerebral hemorrhage (ICH) is the most common type of hemorrhagic stroke, accounting for 10–20% of all strokes (1), with a global incidence of 24.6 per 100,000 person-years (2). Spontaneous ICH primarily results from either hypertensive microangiopathy or cerebral amyloid angiopathy (CAA) (3), which are likely to produce varied phenotypes. Hypertensive ICH likely occurs in deep brain structures while CAA-related ICH generally occurs in lobar locations (2). Regardless of the cause, ICH is associated with poor outcomes that include early mortality (2, 4) and the loss of functional independence (2).

Cognitive Impairment (CI) commonly coexists with ICH. The majority of ICH patients exhibit acute phase CI, with impairments reported in up to 84% of patients (5). While the immediate post-ICH cognitive effects and the potential for long-term CI (6) are broadly recognized, several evidence gaps persist. The trajectory of post-ICH CI is poorly characterized and demonstrates considerable variability. Some ICH patients experience favorable recovery after an acute cognitive decline while others exhibit persistent or worsening CI (7). The significant contribution of cognitive function toward quality of life among ICH survivors has driven an increased research focus on post-ICH CI and dementia. With the growing body of literature focused on post-ICH CI, it is important to integrate the available evidence and characterize cognitive function among ICH patients. This systematic review aims to collect and summarize current evidence regarding the risk factors and trajectory of CI after spontaneous ICH, report the strength and validity of study methodologies, and highlight current knowledge gaps in the study of post-ICH CI.

## Methods

### Search Strategy

We adopted a two-stage systematic review approach to comprehensively account for all study types that have reported cognitive function among patients with spontaneous ICH, particularly focusing on post-ICH CI. Articles were to be excluded from consideration if they did not address the occurrence of CI after ICH, focused on animal or tissue models, assessed other/mixed stroke etiologies or traumatic ICH, or assessed a pediatric/neonatal population. First, a literature search within the PubMed and ScienceDirect databases was performed. Searches were performed using the keywords [“intracerebral hemorrhage” OR “ICH”] AND [“cognitive impairment,” OR “cognitive decline” OR “dementia”] to detect both abbreviated and unabbreviated descriptions. Searches were performed using Boolean logic with detection of alternative spellings (e.g., hemorrhage and hemorrhage) enabled. No limits were placed on date of publication, and databases were last accessed on November 3rd of 2020 (PubMed) and October 27th of 2020 (ScienceDirect). To enable inclusion of studies with higher methodological rigor, only peer-reviewed clinical trials, observational studies, and case series were included. After accounting for duplicates, articles were screened for inclusion. Narrative reviews, study protocols, animal studies, histological studies, editorial comments, expert opinions, abstracts lacking a full-text publication, and papers written in a non-English language were removed from initial assessment. Abstracts were then reviewed for the concepts related to ICH and CI or dementia and rejected if they failed to mention one or both. Abstracts were also rejected at this stage if they specified a pediatric population or addressed subarachnoid/traumatic ICH, ischemic stroke, or transient ischemic attacks. Abstracts that included non-specific terminology (e.g., “stroke” instead of specifying ischemic or hemorrhagic etiologies) were included for further review. Full texts were then reviewed for all articles that did not meet the rejection criteria. Articles that made use of a mixed stroke cohort without delineation were excluded, as were studies that only assessed CI before or independent of ICH. Data were independently extracted from included studies and, when available, included study design, sample size, country of origin, follow up time, neuropsychological assessments and thresholds, imaging methods and assessed markers, other assessments, clinical/social factors and medications, overall rates of CI, factors significantly associated with CI, and other outcome notes. No separate review protocol has been registered or published for this study.

To supplement the initial review, a secondary snowball review was performed based on the articles returned by the original search methodology; as has been recommended in published literature (8). Publications were screened for discussions that linked CI to ICH, and cited articles were examined for information regarding post-ICH CI in the same manner as articles obtained by database search.

### Data Extraction

Articles were assessed for methodology, neuropsychological assessments, imaging findings, other clinical assessments, other factors, and study results, and data were extracted within these categories. Recorded methodology included the study design (cross-sectional, clinical trial, case-control, cohort, etc.), country of origin, number of ICH patients, and follow-up times. Cognitive assessment details included testing methods and thresholds used to define CI and/or dementia. Imaging measures included the applied modalities and features assessed. “Other clinical assessments” included assessments which address factors other than CI (stroke severity, depression, disability, etc.). Notably, stroke laterality was variably assigned between “Imaging” and “Other Clinical Assessments” depending on whether assessment was linked to imaging within the assessed document. Data retained as “other factors” included clinical risk factors and/or laboratory values (comorbidities, etc.), social risk factors (smoking history, alcohol consumption, education, race, etc.), and medications. Each study was assessed for the observed or reported CI percentage at follow-up, hazard rates, impairment domains, other listed cognitive outcomes, factors associated with CI, and other outcome notes. Impairment percentage was calculated based on patient numbers when not independently reported for ICH patients. Data were independently extracted by the lead author (TP) and checked for quality and validated by other authors (JM, AP, and FV). All data for this review was stored within an internal database for reference and reproducibility.

### Risk of Bias Assessment

The risk of bias for each study was assessed following the Agency for Healthcare Research and Quality's (AHRQ) bias assessment tool, classifying risk as low, medium high, or unclear (9). Briefly, the AHRQ tool provides a design-specific method for assessing bias in scientific studies. Specific attention is paid to the recruitment, allocation, and retention of individuals within study groups, protocol consistency, use of outcome measures, and the complete reporting of outcomes.

### Statistical Methods

Sample sizes and proportions of ICH patients with CI across the follow-up timeline were extracted for assessment. Follow-up times were also recorded and are reported (median, mean, or set times). The maximum, minimum, and average reported CI proportion (measured as a percentage) was determined at each follow-up time, and these values were used to assess the overall trajectory of CI after ICH. Given the heterogeneity and lack of information about ICH clinical phenotypes and variety of CI assessment methods employed across a spectrum of post-ICH timepoints no meta-analytic methods were applied for data pooling and summarization. Detailed descriptive accounts of study characteristics, post-ICH CI, assessment methods, imaging findings, and clinical and socio-demographic factors are provided.

## Results

**Figure 1:**
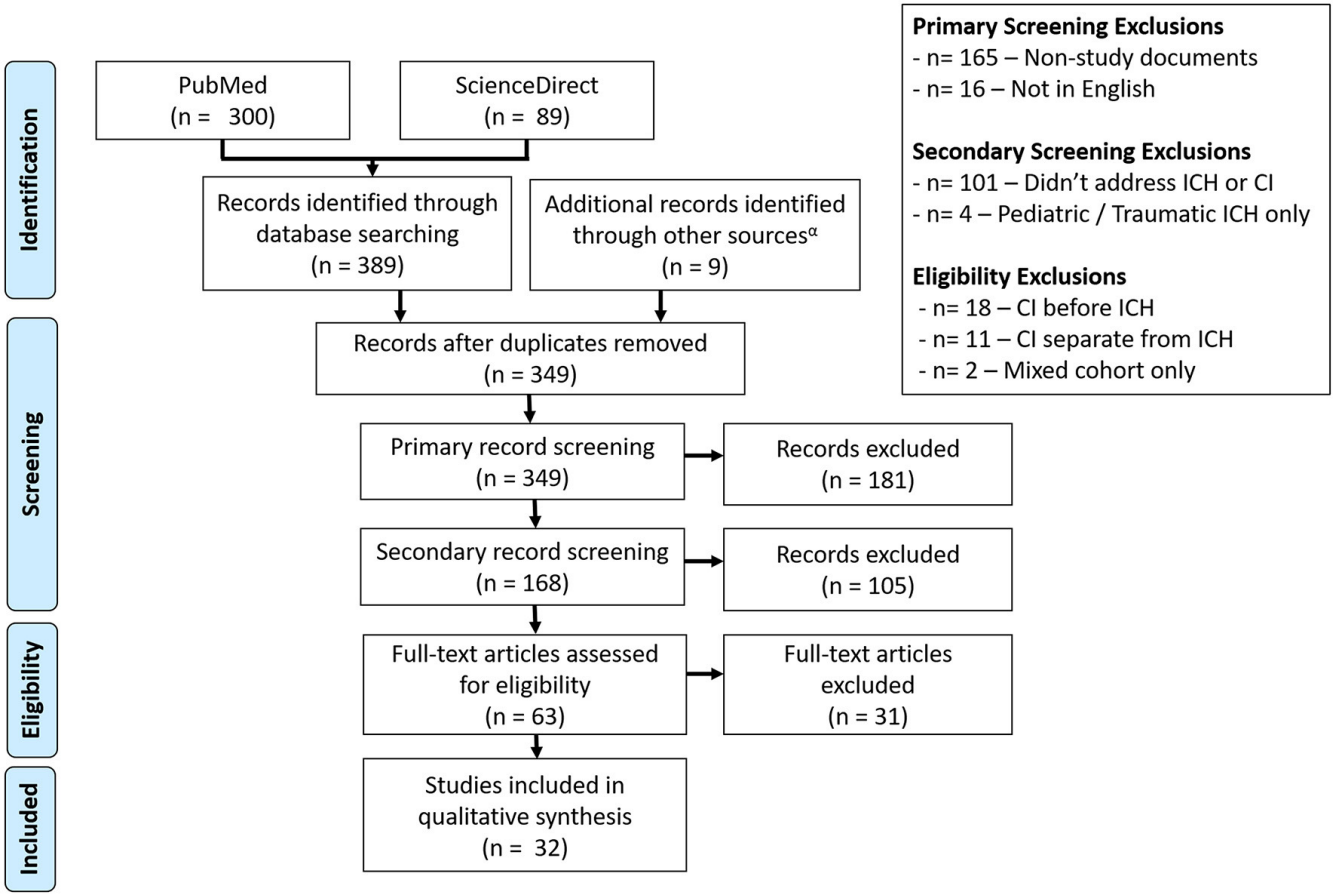
Consort diagram for the selection of studies. A total of 32 studies were selected from the Science Direct and PubMed databases, with screening performed to remove articles that did not address post-ICH CI or non-spontaneous adult ICH cases.

Following the outlined search criteria and syntax 300 articles were identified from the PubMed database with additional 89 records obtained from the ScienceDirect database. Forty-nine overlapping records were removed. Additional articles were removed for the following reasons: were a protocol, opinion review, textbook chapter, meeting proceedings, or other unrelated document type (*n* = 165), were not available in English (*n* = 16), did not address CI and/or spontaneous ICH in an adult population (*n* = 101), assessed pediatric or traumatic ICH (*n* = 4), assessed CI before ICH (*n* = 18), assessed the CI separately from ICH (*n* = 11), or assessed a mixed stroke cohort (*n* = 2). This yielded 23 total articles obtained from literature review. After identifying more sources of interest within the references of conceptually related reviews, a secondary snowball literature was performed based on the reviews identified from initial search. This search yielded nine additional sources, which were combined with the initial search results to form the final set of works for review (*n* = 32). An overview of the literature selection process is depicted in Figure 1. Of the 32 selected studies, 19 (59.4%) were observational cohorts, 8 (25.0%) were case-control studies and 3 (9.4%) were cross-sectional analyses. Additionally, data from 1 (3.1%) randomized control trial (RCT) and case series were included. Studies cumulatively included 22,631 ICH patients. The content of reviewed studies can be found in Tables 1–3, with cohort studies presented in Table 1, case-control studies in Table 2, and other studies in Table 3. Assessed risks of bias for each study are presented in Table 4. Of the 32 assessed studies, 31 (96.9%) studies were found to be of “acceptable” risk of bias. The lone exception was a limited case series and of unclear bias due to the limited details presented. Fourteen studies (43.8%) excluded patients with severe aphasia. Of these, 11 studies excluded 62 patients who could not communicate or follow directions (11, 14, 21–23, 25, 27, 29, 31, 33, 38) while the three remaining studies excluded aphasia without providing the number excluded (10, 15, 16). Eleven studies (34.4%) only included living patients and did not account for potential survivorship bias within the studied ICH population (12, 14, 21, 22, 24, 26, 30, 33, 34, 36, 38). Though the overall bias risk was acceptable, one study (3.2%) also had a significant difference in follow-up times between the ICH and non-ICH study groups (30).

**Table 1:** Summary of methods and findings from cohort studies.

**References**	**Method** **(A) Design** **(B) Cohort size** **(C) Cohort Nationality** **(D) Assessment/follow-up times**	**Neuropsychological assessment** **(A) Cognitive tests or tested domains** **(B) Impairment Criteria** **(C) Treatment of pre-existing impairment**	**Imaging** **(A) Modality** **(B) ICH markers** **(C) Other factors**	**Other assessments**	**Other factors** **(A) Clinical/laboratory risk factors** **(B) Social risk factors** **(C) Medication**	**Results** **(A) Rate of impairment at follow-up** **(B) Factors for impairment** **(C) Other notes**
Aam et al. (7)	(A) Cohort study (B) *n* = 53 [intracerebral hemorrhage (ICH)] (C) Norwegian (D) 3 months, 18 months	(A) National Institute of Neurological Disorders and Stroke and Canadian Stroke Network (NINDS-CSN) battery; Montreal Cognitive Assessment (MoCA)/Telephone—MoCA (T-MoCA), Trail Making Task A and B (TMT-A and TMT-B), Verbal Fluency Letter Test, Consortium to Establish a Registry for Alzheimer's Disease (CERAD) battery, Global Deterioration Scale (GDS) (B) mild neurocognitive disorders (NCD) criteria, DSM-5, Global Deterioration Scale (GDS) 4–7 for premorbid dementia (C) Excluded	–	National Institute of Health Stroke Scale (NIHSS), stroke laterality, Barthel Index (BI), modified Rankin Scale (mRS)	–	(A) Impairment probability: 0.66 at 3 months, ~0.53 at 18 months (B) – (C) Language impaired at 3 months, recovered at 18. Recovery non-significant. Study underpowered
Altieri et al. (10)	(A) Cohort study (B) *n* = 4 (C) Italian (D) annual for 4 years	(A) Auditory Verbal Learning Test, Rey Complex Figure, Corsi Block-Tapping Test, Stroop Test, Rey Complex Figure, Trail Making Test, Wisconsin Card Sorting, Colored Progressive Matrices, Controlled Oral Word Association, Animal Naming, Aachner Naming subtest (B) ICD-10 diagnoses; impairment of memory and one more domain. Etiological diagnosis following NINCS-ADRDA, National Institute of Neurological Disorders and Stroke—Association Internationale pour la Recherche et l'Enseignement en Neurosciences (NINDS-AIREN) criteria (C) Excluded	–	–	–	(A) 1/4 patients developed dementia (B) Not explored in ICH (C) –
Barba et al. (11)	(A) Cohort study (B) *n* = 29 (C) Spanish (D) 3 months	(A) Spanish Informant Questionnaire on Cognitive Decline in the Elderly (IQCODE), Short Portable Mental Status Questionnaire (SPMSQ), neuropsych battery (mental status questionnaire, Mini-mental State Evaluation (MMSE), visual and hearing reaction time, bell test, verbal fluency, picture recognition, word learning, logic memory, block test, naming, token test, similarities, and lawton-brody scale), and clinical interview (B) Clinical interview for diagnosis according to DSM-IV, NINDS-AIREN criteria for vascular dementia (C) Included as factor	–	–	–	(A) 8/29 (27.5%) post-stroke dementia in hemorrhagic stroke (B) Not explored in ICH (C) –
Benedictus et al. (12)	(A) Cohort study (B) *n* = 233 (C) France (D) 6 Months, 12 months, annual	(A) MMSE, premorbid IQCODE (B) IQCODE ≥ 64 for dementia, ≥53 for impairment (C) Excluded dementia, impairment treated as a factor	(A) CT and MRI (B) Lobar/nonlobar/unknown ICH, cortical atrophy, white matter hyperintensities (WMH) (Fazekas scale 0–1 vs. 2–3), lacune presence, microbleed presence	mRS, Montgomery-Asberg Depression Rating Scale (MADRS)	(A) Previous stroke, transient ischemic attack (TIA), and atrial fibrillation (B) Age, sex, and education (>8 or ≤ 8 years) (C) –	(A) 37% of patients showed decline during follow-up, median decline of 1 MMSE point per year (B) Decline in overall cohort significantly linked to premorbid IQCODE ≥ 53, with marginal significance for age, female sex, previous stroke/TIA, microbleed presence, WMH severity, cortical atrophy severity. Decline in patients without pre-existing impairment linked only to previous and follow-up stroke/TIA, with marginal significance for cortical atrophy (C) Baseline MMSE was 27.
Biffi et al. (13)	(A) Cohort study (B) *n* = 738 (C) American (D) 3 months, 6 months, semiannual	(A) Modified Telephone Interview for Cognitive Status (TICS-m) (B) TICS-m < 20, IQCODE > 3.3 (premorbid exclusion criteria) (C) Excluded	(A) CT and MRI (B) CT-defined white matter disease (WMD), MRI white matter hyperintensities (WMH), MRI cerebral microbleeds (CMBs), lobar/deep/cerebellar/multiple location ICH, ICH volume, intraventricular extension	–	(A) APOE genotype (E2/E3/E4), history of mood disorder, hypertension, ischemic heart disease, atrial fibrillation, diabetes, anxiety disorder, prior lobar ICH, prior nonlobar ICH, and prior ischemic stroke (B) Age, race (European American/African American/Asian American/Hispanic/Other), education (<10/≥10 years) (C) Antiplatelet agents, warfarin, statins, antihypertensive agents, selective serotonin reuptake inhibitors (SSRIs)	(A) 279/738 (39%) developed dementia after ICH. 5.2% ICH recurrence/year. 140/738 (19%) developed dementia within 6 months (EPID), 139/738 (19%) developed after 6 months (DPID) (B) EPID significantly associated with African American race, ICH location, ICH volume, age, APOE E2. DPID significantly associated with age, education level, African-American race, diagnosis of mood disorder, WMD severity, lobar CMB burden, APOE E4 (C) –
Koning et al. (14)	(A) Cohort study (B) *n* = 19 (C) Dutch (D) 3–9 months	(A) Blessed Dementia Scale (BDS) for pre-morbid dementia, Cambridge Cognitive Examination and neuropsych battery (B) DSM-IV criteria for dementia applied, subclassified according to NINDS-AIREN (C) Included	–	–	–	(A) 8/19 ICH patients showed dementia on follow-up (B) Not explored in ICH (C) –
Douiri et al. (15)	(A) Cohort study (B) *n* = 169 (C) English (D) 3 months, annual	(A) MMSE or Abbreviated Mental Test (AMT) in both acute phase and follow up (B) Cutoffs: MMSE = 24, AMT = 8 (C) Not assessed/available	–	–	–	(A) 68/169 impaired (40.2%) at 3 months (B) Not explored in ICH (C) –
Gong et al. (16)	(A) Cohort study (B) *n* = 141 (C) Chinese (D) 2 weeks (early impairment), 6 months (late impairment)	(A) MoCA, short-MoCA (B) Impairment: MoCA < 26, short-MoCA < 7 (C) Excluded	(A) MRI and CT/MR angiography (B) WMH severity (Fazekas), ICH location (lobar/nonlobar), hematoma size, intracranial arterial stenosis	NIHSS	(A) Admission sBP, hypertension, diabetes mellitus, excessive smoking, excessive drinking, fever, constipation, hyperglycemia, hyponatremia, C-reactive protein (CRP), red blood cell count (RBC), white blood cell count (WBC), hemoglobin, hematocrit, mean corpuscular volume, mean corpuscular hemoglobin, mean corpuscular hemoglobin concentration, red cell distribution width, total protein, albumin, alanine aminotransferase, aspartate aminotransferase, alanine aminotransferase/aspartate aminotransferase ratio, blood oxygen urea, creatinine, estimated glomerular filtration rate, fasting blood glucose, low density lipoprotein, homocysteine (B) Age, sex, and education (≤ 9/>9 years) (C) –	(A) 106/141 (75.2%) with acute stage impairment. 57/90 (63.3%) surviving impaired patients showed CI at 6 months (B) Acute impairment significantly linked to age, dominant-hemisphere hemorrhage, intake sBP, aspartate aminotransferase, ratio of aspartate aminotransferase to alanine aminotransferase, WMH severity, and constipation in univariate analysis. Intake sBP and dominant-hemisphere hemorrhage independently associated with impairment in multivariate assessment. Patients with larger baseline hematoma, lower NIHSS score, dominant hemisphere or lobar ICH, and higher mean corpuscular volume or mean corpuscular hemoglobin showed worse recovery at 6 months. Age and education marginal significance for recovery. Multivariate results showed association with dominant hemisphere hemorrhage, lobar ICH, education, and mean corpuscular volume (C) Survivors with recovery more likely to have better baseline visuospatial/executive, language, attention, and orientation scores.
Greenberg and Charidimou (17)	(A) Cohort Study (B) *n* = 94 (C) American (D) semiannual	(A) Caregiver telephone interview (B) Impairment defined by presence of deficits in memory or other areas relative to 5–10 year baseline (C) Included as factor	(A) MRI, return scans after 12, 18 months (B) Hemorrhage size (micro ≤ 5 mm, macro > 5 mm), location	mRS	(A) Hypertension, Cognitive Impairment, Previous ICH, APOE Genotype (E3/E2 or E4) (B) Age (≥ 75/ < 75), Sex (C) –	(A) 53 survived index event without immediate occurrence/history of death or cognitive impairment. 19/53 became cognitively/functionally impaired or died within a mean of 27.9 months (B) Greater hemorrhage burden at baseline increased hazard. Baseline hemorrhages were significantly associated even in non-recurrent cases and the presence of new hemorrhages at follow-up was not predictive of decline (C) –
Griauzde et al. (18)	(A) Cohort study (B) *n* = 142 (C) American (D) 90 days	(A) Premorbid IQCODE, Modified Mini-mental State Evaluation (3 MSE) (B) 3 MSE < 80 (C) Included	–	NIHSS, activities of daily life (ADL), instrumental activities of daily life (IADL), stroke-specific quality of life, Glasgow Coma Scale (GCS),	(A) Pre-ICH mRS (0–1/2–3/4+), atrial fibrillation, diabetes mellitus, hypertension, stroke history, body mass index (B) Age, ethnicity (Mexican American/non-Hispanic White), sex, marital status (married or living together/single/widowed/divorced), Education (Less than high school, high school, some college, college or more), Health insurance, history of smoking, “Do Not Resuscitate” (DNR) orders (C) –	3 MSE data available for 79/107 patients (B) Outcome 3 MSE scores were significantly worse in Mexican American patients (C) –
Ihle-Hansen et al. (19)	(A) Cohort study (B) *n* = 16 (C) Norwegian (D) 12 months	(A) MMSE, Clock Drawing Test, TMT-A and -B, repeated IQCODE (B) Pre-existing cognitive impairment - IQCODE > 3.44. Dementia diagnosed according to ICD-10, mild cognitive impairment defined according to Winblad et al. (20) (C) Excluded	–	–	–	(A) 7/16 impaired (4 with vascular dementia (VaD), 3 with mixed pathology; 43.75%) (B) Not explored in ICH (C) –
Koivunen et al. (21)	(A) Cohort study (B) *n* = 249 (C) Finnish (D) 9.7 years	(A) MoCA (B) Mild impairment: MoCA 18–26, moderate: MoCA 10–17, severe: <10 (C) Not assessed/available	(A) CT (B) Hematoma volume, ICH location, intraventricular extension, multiple hemorrhages, hydrocephalus, herniation	Beck Depression Inventory (BDI), Hospital Anxiety and Depression Scale, Pain Anxiety Scale, Brief Pain inventory (0, 1–48, 49–72, 73–120), mRS	(A) – (B) Age (16–29/30–39/40–49) (C) –	(A) – (B) MoCA scores significantly linked to age and mRS score, increasing with lower age and disability (C) Median age of 50.3, median follow up time of 9.7 years.
Maduriera et al. (22)	(A) Cohort study (B) *n* = 57 (C) Portuguese (D) 3 months	(A) MMSE, NINDS-AIREN battery (B) MMSE < 16 (no schooling), MMSE < 23 (1–10 years schooling), MMSE < 28 (11+ years schooling). Other tests: > 2 SDs below mean (mild CI), > 3 SDs below mean (moderate CI), > 4 SDs below mean (severe CI) (C) Excluded dementia	(A) CT/MR (B) ICH location (Cortical/Subcortical), ICH laterality	Blessed Dementia Scale, Hamilton Depression Rating Scale (≥13), mRS (>2)	(A) Hypertension, diabetes mellitus, atrial fibrillation, previous stroke, acute aphasia (B) Age, gender, education (years), alcohol consumption (C) –	(A) – (B) Patients with no cognitive deficits had significantly higher education levels (C) –
Moulin et al. (23)	(A) Cohort study (B) *n* = 218 (C) French (D) 6 months, 12 months, annually	(A) Premorbid IQCODE, MMSE, NINDS-CSN VCI battery (B) IQCODE between 53 and 63 for impairment, MMSE < 27, dementia diagnosed based on National Institute on Aging—Alzheimer's Association (NIA-AA) criteria for all-cause dementia. Impairment diagnosed by history (C) Excluded dementia, impairment treated as a factor	(A) CT and MRI (B) ICH location (lobar/nonlobar/undetermined), ICH volume, cortical atrophy (0–4), leukoaraiosis (0–3), Cortical Superficial Siderosis (focal/disseminated), WMH (Fazekas 0–3), lacune presence, microbleeds [Brain Observer Microbleed Scale (BOMBS)]	Premorbid mRS (dependency > 2), NIHSS	(A) Previous stroke or TIA, ischemic heart disease, atrial fibrillation, hypercholesterolemia (B) Age, sex, level of education (>8/ ≤ 8 years), smoking, alcohol intake (C) –	(A) 63 (28.9%) developed new-onset dementia, 69 (31.7%) died. 29/69 deceased developed dementia prior to death. Incidence rate of 14.2% (1 year), 19.8% (2 years), 24.5% (3 years), 28.3% (4 years) (B) Age, previous stroke, pre-existing impairment, NIHSS at onset, lobar location, leukoaraiosis score (≥3), and occurrence of secondary stroke/TIA during follow up were baseline characteristics significantly linked to new-onset dementia. Microbleed number (>5), disseminated superficial siderosis, atrophy score, and age significantly associated in MRI characteristic model. Baseline characteristics associated with post-lobar-ICH cognitive impairment (CI) (age adjusted): Leukoaraiosis score (≥3), NIHSS, cortical atrophy score, pre-existing CI, older age. MRI characteristics associated with post-lobar ICH CI (age-adjusted): disseminated superficial siderosis, cortical atrophy score, previous hemorrhage, older age (C) Median follow-up of 6 years, median interval of 12 months between ICH and dementia diagnosis
Nakase et al. (24)	(A) Cohort study/comment (B) *n* = 266 (C) Japanese (D) Acute phase	(A) – (B) DSM-IV criteria for cognitive impairment (C) Not assessed/available	–	NIHSS (admission/discharge)	(A) WBC, CRP, estimated glomerular filtration rate, hypertension, hyperlipidemia, diabetes mellitus (B) Age, gender (C) –	(A) 49/256 cognitively healthy individuals developed impairment after ICH (B) Impaired group had significantly higher NIHSS at admission and discharge, higher CRP and WBC (C) CRP and WBC indicate inflammation
Planton et al. (25)	(A) Cohort study (B) *n* = 40 (C) French (D) 3 months	(A) Premorbid IQCODE, MMSE, Free and Cued Selective Reminding Test (FCSRT), Delayed Matched Sample test, FAB, TMT-A, Stroop test, Digit Span Forward and Backward, Mahieux's test (B) IQCODE ≥ 3.4 for cognitive decline, VAS-COG criteria (mild = 1+ impaired domain within 1–2 SDs of normal, major = 2+ impaired domains 2+ SDs below norm) (C) Excluded	(A) MRI (B) White matter hyperintensities (Fazekas), number of cerebral microbleeds (BOMBS), presence of superficial cortical siderosis, ICH volume, ICH laterality	MADRS, BDI, NIHSS onset, NIHSS at follow-up, mRS at follow-up	(A) APOE E2, APOE E4, hypertension, diabetes, dyslipidemia, obesity, previous stroke (B) Age, gender, education (years), delay from ICH to scan, delay from ICH to assessment, smoking, (C) Antiplatelet use	(A) Final cohort had 20 patients with deep ICH and 20 with lobar ICH (B) ICH volume, laterality, presence of CSS, presence of strictly lobar microbleeds, hypertension, and dyslipidemia significantly different between deep and CAA-related ICH groups. CAA and deep ICH groups had significantly worse MMSE scores, FCSRT, Stroop denomination and reading times, TMT-A times, and TMT-A times than healthy controls. Deep ICH alone had significantly worse FAB scores and TMT-A error rates, while CAA-ICH had worse scores in the backward digit span and naming tasks (C) Median delay for testing was 4 months after ICH.
Tang et al. (26)	(A) Cohort study (B) *n* = 23 (C) Chinese (D) 3 months	(A) IQCODE, MMSE (B) Post stroke dementia diagnosed based on DSM-IV criteria for vascular dementia (C) Included as factor	–	–	–	(A) 4/23 ICH patients developed post-stroke dementia (17.3%) (B) Not explored in ICH (C) –
Tveiten et al. (27)	(A) Cohort study (B) *n* = 44 (C) Norwegian (D) 3.8 years (median)	(A) MoCA (B) MoCA ≤ 23, MoCA ≤ 25 (C) Not assessed/available	(A) CT (B) ICH location (lobar/deep/brainstem/cerebellum), ICH volume, intraventricular extension, leukoaraiosis score (0–4)	GCS (3–4/5–12/14–15)	(A) Diabetes mellitus, coronary heart disease, atrial fibrillation, hypertension (B) Age (years), sex, low education (listed as elementary school only, 7 years up to 1960, or 9 years thereafter), smoking (C) –	(A) MoCA assessed in 44 patients out of original cohort of 51. 27/44 (61.4%) fell below ≤ 23 cutoff, 31/44 (71%) fell below ≤ 25 cutoff (B) Univariate analysis found age, sex, low education, lobar ICH, and leukoaraiosis score associated with MoCA ≤ 23, while multivariate analysis found significance for age and lobar ICH location. The same factors were found for the MoCA ≤ 25 threshold (C) –
Xiong et al. (28)	(A) Cohort study (B) *n* = 97 (C) American (D) 6 months	(A) Standardized notes from clinical follow-up (B) Mild CI determined when patients had impaired cognition but preserved independence, dementia defined as cognitive decline that interfered with work or daily activities. Followed NIA-AA workgroup criteria (C) Excluded dementia, impairment treated as a factor	(A) CT (B) ICH volume, intraventricular extension, lacunes, CMB number, CMBs ≥ 5, WMH volume, total brain volume, WMH (Fazekas), enlarged perivascular spaces, focal CSS burden, disseminated CSS burden, global cortical atrophy, CSVD score, CSVD score binarized (≥3, <3)	–	(A) History of hemorrhagic stroke, hypertension, diabetes, hypercholesterolemia, pre-existing MCI (B) Age (years), sex, education (none, 1–6 years, 7–9 years, 10–13 years, 14+ years) (C) –	(A) 25/97 patients (25.8%) without early dementia developed dementia during the follow-up time (incidence rate of 37.4%) (B) Older age, lower education, pre-existing MCI, lobar CMBs ≥ 5, WMH burden, disseminated CSS, higher CCSVD score were associated with dementia conversion in univariate analysis. Separate models created for multivariable analysis for imaging and clinical/social factors. Only MCI history associated with dementia conversion in clinical/social factors, while disseminated CSS and WMH burden independently predicted conversion in the neuroimaging model. Sensitivity analysis combining MCI status with disseminated CSS and WMH burden found all factors to be predictive of conversion. MCI history and CSVD score for CAA ≥ 3 predicted conversion in backwards stepwise model (C) Mean follow-up time of 2.5 years,

**Table 2:** Summary of methods and findings from case-control studies.

**Reference**	**Method** **(A) Design** **(B) Cohort size** **(C) Cohort nationality** **(D) Assessment/follow-up times**	**Neuropsychological assessment** **(A) Cognitive tests or tested domains** **(B) Impairment criteria** **(C) Treatment of pre-existing impairment**	**Imaging** **(A) Modality** **(B) ICH markers** **(C) Other factors**	**Other assessments**	**Other factors** **(A) Clinical/laboratory risk factors** **(B) Social risk factors** **(C) Medication**	**Results** **(A) Rate of impairment at follow-up** **(B) Factors for impairment** **(C) Other notes**
Arauz et al. (29)	(A) Case-control study (B) *n* = 14 (ICH) (C) Mexican (D) 3 months	(A) Pre-stroke Informant Questionnaire on Cognitive Decline in the Elderly (IQCODE); Cognitive Abilities Screening Instrument, Clinical Dementia Rating scale, California Verbal Learning Test, Wechsler Adult Intelligence Scale, Controlled Word Association Test, Executive Interview Test, Executive Clock Drawing Task (B) Canadian criteria for vascular cognitive impairment (VCI) without dementia, DSM-4 dementia criteria (C) Excluded	–	NIH Stroke Scale (NIHSS); stroke laterality, subcortical/cortical, and anterior/posterior circulation; Barthel Index (BI), modified Rankin Scale (mRS)	(A) Homocysteine, hypercholesterolemia, transient ischemic attacks (TIAs), diabetes mellitus, hypertension (B) Smoking, alcohol, age, sex, education (years), education (0–9 vs. 10–17 years) (C) –	(A) 2 cases of dementia (14%), 10 cases of cognitive impairment (71%) (B) No factors significantly associated to outcome, VCI contributed to poorer mRS (C) –
Banerjee et al. (5)	(A) Case-control study (B) *n* = 187 (C) England (D) 12 days	(A) Premorbid function, current intellectual function, verbal and visual memory, naming, visuospatial perception, information processing speed, executive function (B) Performance at or below 5th percentile for any domain; executive: failure on 2+ standardized tests, intellectual functioning; difference of 10+ points between verbal or performance IQ measures (C) Not assessed/available	(A) CT and MRI (B) Lobar/non-lobar ICH, probable CAA (Boston criteria),	Stroke laterality	(A) – (B) Age, sex, and years of education (C) –	(A) 84% impaired in 1+ domain, 65% in 2+ domains. Non-verbal IQ, processing speed, and executive function most commonly disrupted. Naming, visuospatial processing, and verbal memory less common (B) – (C) Lobar ICH more likely to be older, female, affected in 2+ domains. Probable CAA patients were significantly older and had more bilateral hemorrhages.
Corraini et al. (6)	(A) Case-control study (B) *n* = 16,723 (C) Danish (D) –	(A) None stated (B) ICD-10 dementia diagnosis (C) Not assessed/available	–	–	–	(A) 45% of ICH patients did not survive for 3 months. Hazard ratio of 2.7. Rates: 3–12 months: 20.7, 1–10 years: 13.6, 10–30 years: 10.7 (B) Hemorrhagic stroke had higher risk of dementia than ischemic (C) Records collected from 30 year period (1982–2013). Stratified into 3–12 months/1–10 years/10–30 years
Nakano et al. (30)	(A) Case-control study (B) *n* = 5 (C) Japanese (D) 4.6 years (mean to first assessment), follow-up during 4.8 year period.	(A) Mini-mental State Evaluation (MMSE), Hasegawa Dementia Rating Scale-revised (HDS-R), Frontal Assessment Battery (FAB) (B) MMSE ≤ 23 for post-stroke dementia. Conversion defined by MMSE drop of 4+ points (C) Excluded	(A) MRI and endothelial function scan (B) Reactive hyperemia index, augmentation index, periventricular hyperintensity severity (Fazekas), deep white matter hyperintensity (Fazekas), microbleed number	Geriatric Depression Scale (GDS), Apathy Scale (AS),	(A) Time from first-ever stroke, Body mass index (BMI), abdominal circumference, hypertension, dyslipidemia, diabetes, atrial fibrillation, ischemic heart disease, (B) Age, gender, education (years) (C) –	(A) 0/5 ICH patients developed post-stroke dementia during the follow-up period (B) – (C) Limited ICH population
Nys et al. (31)	(A) Case-control study (B) *n* = 17 (C) Dutch (D) within 3 weeks of event, no follow-up	(A) Short IQCODE, 7–domain neurocognitive assessment (Raven Advanced Progressive Matrices and Similarities, Digit Span, Rey Auditory Verbal Learning Test, Brixton Spatial Anticipation Test, Visual Elevator, Letter Fluency, Judgement of Line Orientation, Test of Facial Recognition, Rey-Osterrieth Complex Figure, Corsi Block Span, Token Test, Boston Naming Test, Star Cancellation) (B) IQCODE = 3.6 (C) Excluded	–	–	–	(A) 14/17 patients impaired in 2+ domains, 3/17 unimpaired (B) Not explored in ICH (C) –
Qureshi et al. (32)	(A) Case-control study (B) *n* = 23 (C) American (D) Annual	(A) MMSE (B) – (C) Not assessed/available	(A) MRI (B) ICH volume, ICH laterality, small infarcts, T1 basal ganglia infarcts, large infarcts, Focal brain atrophy, perivascular spaces (mild/marked), cortical infarcts, white matter brainstem lesions (minimal/moderate or severe)	–	(A) Hypertension, diabetes mellitus, cognitive heart failure, previous stroke, serum cholesterol, BMI, AND sBP (B) Age, sex, ethnicity (White/African American), smoking (never/former/current) (C) Warfarin, aspirin	(A) MMSE scores higher in ICH group than control at 3 year follow up, however scores were similar at 5 year follow-up (B) Factors not significantly different between persons with and without ICH, no impairment for assessment at 5-year follow-up (C) Focused on asymptomatic ICH. Median follow-up time for sample was 82.6 months.
Su et al. (33)	(A) Case-control study (B) *n* = 312 (C) Taiwanese (D) within 6 months	(A) MMSE, Battery drawn from other tests–Digit Span, Visual memory span, Wechsler Memory Scale-Revised serial-seven subtractions (from MMSE), block design (from Wechsler Adult Intelligence Scale–Revised), Hooper Visual Organization test, Spatial relationships and Form constancy (from Test of Visual-Perceptual Skills), Time and place (from MMSE), 15-s delayed recall, Form D (from Benton visual retention test), Verbal memory scale, Receptive Speech and Expressive speech (from Luria-Nebraska Neuropsychological Battery), Wisconsin Cart Sorting Test (B) Impairment defined as a *z*-score of 2+ SDs below control mean (C) Excluded	(A) CT or MRI (B) ICH laterality, ventricular extension	Initial GCS	(A) Craniotomy (B) Age (years), education (years), (C) –	(A) MMSE scores were significantly lower in the ICH population. Between-group differences found between ICH and healthy individuals in attention, memory, visuospatial function, and executive function. Visuospatial function and memory had greatest rates of significant impairment. 90% of patients with MMSE scores <24 had impairment in 6–7 domains. Cognitive test predictors were able to discriminate patients from controls with 95.5% accuracy (B) Stroke laterality correlated with all domains except for attention and memory, mild correlation between admission GCS and executive function. Right ICH performed worse in visuospatial and executive function, patients with left ICH performed worse in language and memory (C) ICH population had significantly fewer females than healthy controls

**Table 3:** Summary of methods and findings from other study types.

**Reference**	**Method** **(A) Design** **(B) Cohort size** **(C) Cohort nationality** **(D) Assessment/follow-up times**	**Neuropsychological assessment** **(A) Cognitive tests or tested domains** **(B) Impairment criteria** **(C) Treatment of pre-existing impairment**	**Imaging** **(A) Modality** **(B) ICH markers** **(C) Other factors**	**Other assessments**	**Other factors** **(A) Clinical/laboratory risk factors** **(B) Social risk factors** **(C) Medication**	**Results** **(A) Rate of impairment at follow-up** **(B) Factors for impairment** **(C) Other notes**
Espinosa del Pozo et al. (34)	(A) Cross-sectional Study (B) *n* = 9 (C) Ecuadorian (D) –	(A) Mini-mental State Evaluation (MMSE), Ascertain Dementia Eight-Item Informant Questionnaire (AD8) (B) MMSE: Normal > 23; 19–23, mild cognitive impairment; 10–18, moderate impairment, <10 severe impairment; AD8: 0–1 normal, ≥2 impaired (C) Not assessed/available	–	Mini Nutritional Assessment	–	(A) 6/9 ICH patients showed moderate impairment, 3/9 unimpaired in MMSE. 5/8 impaired, 3/8 unimpaired in AD8 (B) Intracerebral hemorrhage significantly associated with lower MMSE scores, not associated with AD8 results (C) 2 with good nutritional status, 4 at nutritional risk, 3 malnourished
Griauzde et al. (18)	(A) Cross-sectional study (B) *n* = 78 (C) French (D) 40 months	(A) premorbid National Adult Reading Test, MMSE, Montreal Cognitive Assessment (MoCA), Mattis Dementia rating scale (B) Dementia diagnosed using DSM-IV; NINDS-AIREN and National Institute of Neurological and Communicative Disorders and Stroke and the Alzheimer's Disease and Related Disorders Association (NINCDS-ADRDA) criteria for dementia; CI defined as performance at or below 5th percentile for any domain (C) Included	(A) CT and MRI (B) Hemorrhage location (deep/lobar/infratentorial), laterality, hemorrhage volume, Cerebral amyloid angiopathy (CAA), white matter abnormalities	Goldberg Anxiety Scale, Hamilton Rating Scale for Anxiety, Montgomery–Asberg Depression Rating Scale (MADRS), Barthel Index (BI), Instrumental Activities of Daily Life (IADL), Modified Rankin Scale (mRS)	(A) Hypertension, alcoholism, history of hemorrhage, history of stroke/TIA, and dementia (B) – (C) –	(A) Persistent neurological deficits observed in 51/78 patients at follow up (39 motor, 27 sensory, 33 gait, and 12 aphasic). Impairment found in 37/78 patients with full battery of results, dementia observed in 18/78 patients (B) Initial signs of headache, delirium, and vigilance deficits, hemorrhage volume, and laterality significantly associated with dementia (C) –
Miller et al. (35)	(A) Case series (B) *n* = 7 (C) Scottish (D) 30 months	(A) Not described (B) – (C) Included	(A) CT (B) White matter hyperintensity (WMH) assessment, lobar hemorrhage, intraventricular extension	–	(A) Hypertension (B) – (C) –	(A) 3/7 patients died within 2 weeks of onset. All patients surviving patients showed some evidence of progressive cognitive impairment, despite no previous history (B) 5/7 showed periventricular white matter lucencies, all had white matter disease. 5/7 had lobar stroke with intraventricular extension (C) Focused on 7 patients with CAA-related ICH.
Sakai et al. (36)	(A) Cross-sectional study (B) *n* = 474 (C) Japanese (D) –	(A) None, impairment indicated by record (B) – (C) Not assessed/available	–	–	–	(A) Impairment found in 208/442 (47.1%) after first episode, 41/77 (53.2%) after second episode, 15/24 (62.5%) after third episode, 5/9 (55.6%) after the fourth episode, and 2/2 (100%) after the fifth episode (B) Number of ICH events (C) Retrospective study of medical records focused on CAA-related ICH
You et al. (37)	(A) Randomized clinical trial (B) *n* = 231 (C) International (D) 90 days	(A) MMSE (B) MMSE ≤ 24 for impairment, MMSE ≤ 18 for severe impairment (C) Excluded	(A) CT (B) ICH location (lobar/deep), ICH laterality, hematoma volume, intraventricular extension, perihematoma edema at 72 h, randomized intensive BP lowering	Glasgow coma scale (GCS), NIH Stroke Scale (baseline/24 h/7 days or discharge)	(A) Prior ICH, prior ischemic stroke, acute coronary event, diabetes mellitus, hypertension, systolic BP at presentation, diastolic BP at presentation, mean systolic BP over first 24 h (B) Age, gender (C) Antihypertensive therapy, warfarin, aspirin	(A) 75/231 (32.5%) had MMSE ≤ 24 (B) Impairment associated with older age, female gender, history of ICH or ischemic stroke, higher baseline NIHSS, less intraventricular extension, lower diastolic BP at baseline, and higher mean systolic BP over the first 24 h. Similar results found for MMSE ≤ 18. Multivariable analysis found prior ICH, female gender, older age, higher baseline NIHSS, and higher systolic BP over first 24 h independently linked to impairment (C) –

**Table 4:** Assessment of risk for included studies, following AHRQ guidelines.

**Reference**	**Aam et al. (7)**	**Altieri et al. (10)**	**Arauz et al. (29)**	**Banerjee et al. (5)**	**Barba et al. (11)**	**Benedictus et al. (12)**	**Biffi et al. (13)**	**Corraini et al. (6)**	**Koning et al. (14)**	**Douiri et al. (15)**	**Espinosa del Pozo et al. (34)**	**Garcia et al. (38)**	**Gong et al. (16)**	**Griauzde et al. (18)**	**Greenberg et al. (39)**	**Ihle-Hansen et al. (19)**	**Koivunen et al. (21)**	**Li et al. (40)**	**Madureira et al. (22)**	**Miller et al. (35)**	**Moulin et al. (23)**	**Nakano et al. (30)**	**Nakase et al. (24)**	**Nys et al. (31)**	**Planton et al. (25)**	**Qureshi et al. (32)**	**Sakai et al. (36)**	**Su et al. (33)**	**Tang et al. (26)**	**Tveiten et al. (27)**	**Xiong et al. (28)**	**You et al. (37)**
Overall risk^*^	L	L–M	L–M	L–M	L–M	L–M	L	L	M	L–M	M	L–M	L	L	M	L–M	L–M	M	M	U	L	M	L–M	L–M	M	L–M	M	L–M	L	L	L	L
Selection bias^*^																																
Was the allocation sequence random?	–	–	–	–	–	–	–	–	–	–	–	–	–	–	–	–	–	–	–	–	–	–	–	–	–	–	–	–	–	–	–	U
Was the allocation of treatment adequately concealed?	–	–	–	–	–	–	–	–	–	–	–	–	–	–	–	–	–	–	–	–	–	–	–	–	–	–	–	–	–	–	–	Y
Were participants analyzed within their original groups?	–	Y	–	–	Y	Y	Y	–	Y	–	–	–	Y	Y	Y	–	Y	–	Y	–	Y	–	Y	–	Y	–	–	–	–	Y	Y	Y
Did the study apply inclusion/exclusion criteria uniformly?	Y	Y	–	–	Y	Y	Y	–	Y	Y	Y	Y	Y	Y	Y	Y	Y	–	Y	–	Y	–	Y	–	N	–	N	–	–	Y	Y	–
Were cases and controls selected appropriately?	–	–	Y	Y		–	–	Y	–	–	–	–	–	–	–	–	–	Y	–	–	–	Y	–	Y	–	Y	–	Y	–	–	–	–
Did the recruitment strategy differ across study groups?	N	N	–	–	N	N	N	Y	N	N	–	–	N	N	N	N	N	–	N	–	N	–	N	–	U	–	–	–	N	N	N	–
Does the study account for confounding and modifying variables?	Y	U/N	N	Y	Y	Y	Y	Y	S	Y	S	Y	Y	Y	Y	S	Y	Y	Y	–	Y	S	U	Y	S	Y	N	Y	Y	Y	Y	Y
**Performance bias**																																
Did the study rule out any impact from a concurrent intervention?	N	Y	Y	Y	Y	Y	Y	Y	Y	Y	N	Y	Y	Y	Y	Y	–	Y	Y	–	Y	Y	Y	Y	Y	Y	Y	–	Y	–	Y	Y
Did the study maintain fidelity to protocol?	–	Y	–	Y	Y	Y	Y	Y	Y	Y	–	Y	Y	Y	Y	Y	Y	Y	Y	–	Y	Y	Y	Y	Y	Y	–	Y	Y	Y	Y	Y
**Attrition bias**																																
If attrition was a concern, were missing data addressed?	Y	Y	–	Y	Y	N	Y	Y	N	Y	N	N	Y	Y	Y	Y	N	Y	N	–	Y	N	Y	Y	N	Y	N	N	N	Y	S	Y
**Detection bias**																																
Were follow up times the same between groups?	Y	Y	Y	Y	Y	Y	Y	Y	Y	Y	–	–	Y	Y	Y	Y	U	Y	Y	–	Y	N	Y	Y	Y	Y	–	Y	Y	U	Y	Y
Were the outcome assessors blinded appropriately?	–	N	–	N	U	Y	U	U	Y	U	–	Y	Y	Y	Y	U	U	U	Y	N	Y	N	U	Y	U	U	–	Y	Y	U	Y	Y
Were exposures assessed using valid and reliable measures?	Y	Y	Y	Y	Y	Y	Y	Y	Y	Y	Y	Y	Y	Y	Y	Y	Y	Y	Y	U	Y	Y	Y	Y	Y	Y	Y	Y	Y	Y	Y	Y
Were outcomes assessed using valid and reliable measures?	Y	Y	Y	U	Y	Y	Y	Y	U	Y	Y	Y	Y	Y	U	Y	Y	Y	Y	U	Y	Y	Y	U	Y	Y	Y	Y	Y	Y	Y	Y
Were confounding variables assessed using valid and reliable measures?	Y	Y	Y	Y	Y	Y	Y	Y	Y	Y	Y	Y	Y	Y	N	Y	Y	Y	Y	U	Y	N	Y	Y	Y	Y	Y	Y	Y	Y	Y	–
**Reporting bias**																																
Were the outcomes prespecified and all outcomes reported?	Y/Y	Y/Y	Y/Y	Y/Y	Y/Y	Y/Y	Y/N	Y/Y	Y/Y	Y/Y	Y/Y	Y/Y	Y/Y	Y/Y	Y/N	Y/Y	Y/Y	Y/Y	Y/Y	Y/Y	Y/Y	Y/Y	Y/Y	Y/Y	Y/Y	Y/Y	Y/Y	Y/Y	Y/Y	Y/Y	Y/Y	Y/Y

* *L, Low, M. Medium; H, High; U, Unclear; S, Some; Y, Yes; N, No*.

### Study Characteristics

Among the 32 included studies, 19 (59.4%) publications provided investigations of the various factors that contribute to the development of post-ICH CI. The remaining 13 (40.6%) were either limited in their scope to only describe the observed rate of CI or included heterogenous stroke sub-types, among which ICH was a reported subgroup. Study populations were drawn from the United States (*n* = 5), France (*n* = 4), Japan (*n* = 3), Norway (*n* = 3), the Netherlands (*n* = 2), England (*n* = 2), China (*n* = 2), Taiwan (*n* = 2), 1 each from Finland, Spain, Italy, Scotland, Ecuador, Denmark, Mexico, and Portugal, and 1 international study. Four studies (12.5%) presented fewer than 10 patients (10, 30, 34, 35), of which three studies included ICH only as an sub-group of a larger stroke cohort (10, 30, 34) and the fourth presented as a limited case series (35). In contrast, two studies contained over 1,000 participants each (6, 40). These were large, retrospective national investigations of long-term patient outcomes from Denmark (6) and Taiwan (40). Follow up times were similarly variable, with some studies selecting specific timings to investigate acute and early post-ICH CI/Dementia (<6 months) compared to late post-ICH CI/Dementia (>6 months). Overall, the times for follow-up assessment varied from 12 days to 10 years, with timepoints of 3 months/90 days [10 (29.0%)], 6 months [7 (21.9%)], and 1 year [6 (18.8%)] being the most common. Studies that performed recurrent testing had a minimum test-retest interval of 3 months [1 (3.1%)] and typically assumed annual [5 (15.6%)] or semiannual [2 (6.3%)] testing after the first follow-up. The varying study methods, including the range of follow-up times, are summarized in Table 5.

**Table 5:** The variable testing methods and parameters adopted across studies of post-ICH impairment.

**Follow up times**	***N***	**Cognitive assessments**	***N***	**Imaging factors**	***N***	**Other factors**	***N***
First assessment or stated time		Informant Questionnaire on Cognitive Decline in the Elderly (IQCODE)	7	Imaging	17	NIH Stroke Scale (NIHSS)	8
12 days	1	IQCODE ≥ 64 (dementia)	1	CT	12	Functional assessment	8
2 weeks/14 days	1	IQCODE > 3.5 (impairment)	1	MRI	10	Barthel Index	3
within 3 weeks	1	IQCODE > 3.44 (cognitive decline)	1	CT/MRI	1	Modified Rankin Scale (mRS)	8
3 months/90 days	10	IQCODE ≥ 3.4 (impairment)	1			Activities of Daily Living (ADL)	1
6 months	7	63 ≥ IQCODE ≥ 53 (impairment)	2	Location	11	Blessed Dementia Scale	1
3–9 months	1	IQCODE > 3.3 (dementia)	2	Lobar/nonlobar ICH	3	Depression Assessment	5
12 months	6	Mini-mental State Evaluation (MMSE)	14	Lobar/deep	1	Beck Depression Inventory	2
18 months	1	MMSE < 27 (impairment)	1	Lobar/nonlobar/unknown	2	Montgomery-Asberg Depression Rating Scale (MADRS)	3
30 months	1	MMSE ≤ 24 (impairment)	1	Lobar/deep/cerebellar/multiple	1	Geriatric Depression Scale	1
3 years	1	MMSE < 23 (dementia)	1	Lobar/deep/infratentorial	1	Hospital Anxiety and Depression Scale	1
40 months	1	4-level assessment	1	Lobar/deep/cerebellar/brainstem	1	Hamilton Depression Rating Scale	1
Median 3.8 years	1	MMSE > 23 (normal)		Supratentorial/infratentorial	1	Race/Ethnicity African American/Not Hispanic/Not	3
Within 4.8 years	1	23 ≥ MMSE ≥ 19 (mild impairment)		Cortical/subcortical	1		2
5 years	1	18 ≥ MMSE ≥ 10 (moderate impairment)		Cortical superficial Siderosis (CSS)	3		1
9.7 years	1	MMSE < 10 (severe impairment)		Presence/Absence	1	Previous stroke/ICH/TIA	13
10 years	1	3-level assessment	1	Focal/Disseminated	2	Atrial Fibrillation Diabetes Mellitus	7
		MMSE > 24 (normal)		Volume	9		12
		24 ≥ MMSE > 18		Intraventricular Extension	7	APOE Genotype	3
		18 ≥ MMSE		Microbleeds	5	Education level	13
		Education-based screening assessment	1	White matter hyperintensity (WMH) volume	1	Years	6
		MMSE < 16 (no education)		WMH Severity (Fazekas)	8	>8/≤ 8 years	2
		MMSE < 23 (1–10 years education)		Enlarged Perivascular Spaces	2	<10/≥10 years	1
		MMSE < 28 (11+ years education)		Lacunes	3	≤ 9/>9 years	1
		Montreal Cognitive Assessment (MoCA)	5			4-level	1
		MoCA < 26 (impairment)	2			Less than high school	
		MoCA ≤ 23 (impairment)	1			High school	
		4-level assessment	1			Some college	
		MoCA > 26 (Normal)				College or more	
		26 ≥ MoCA ≥ 18 (mild impairment)				5-level	1
		17 ≥ MoCA ≥ 10 (moderate impairment)				none	
		MoCA< 10 (severe impairment)				1–6 years	
		Ascertain Dementia 8-item (AD8) ≥ 2 (impairment)	1			7–9 years	
		Modified MMSE (3 MSE) < 80	1			10–13 years	
		Modified Telephone Interview for Cognitive Status (TICS-m) < 20	1			14+ years	
		Short MoCA < 7	1			Elementary only, up to 7 years before 1960, 9 years after 1960	1

### Post-ICH Cognitive Impairment Over Time and Contributory Factors

Across studies, the potential impact of preexisting cognitive deficits on post-ICH CI was generally acknowledged. To address this, 13 (40.6%) studies excluded individuals with pre-ICH CI or dementia (7, 10, 13, 16, 19, 22, 25, 29–31, 33, 37, 40), [7 (21.9%)] or included patients with preexisting CI (11, 14, 18, 26, 35, 38, 39), either acknowledging the limitation [4 (12.5%)] (14, 18, 35, 38) or including pre-stroke deficits as a contributory factor for future CI [3 (9.4%)] (11, 26, 39). Three additional studies chose to selectively exclude patients that met the criteria for dementia but included patients with mild impairments, using preexisting dementia as a contributory factor for future deficits (12, 23, 28). The remaining studies [9 (28.1%)] either did not assess or report on preexisting CI (5, 6, 15, 21, 24, 27, 32, 34, 36).

**Figure 2:**
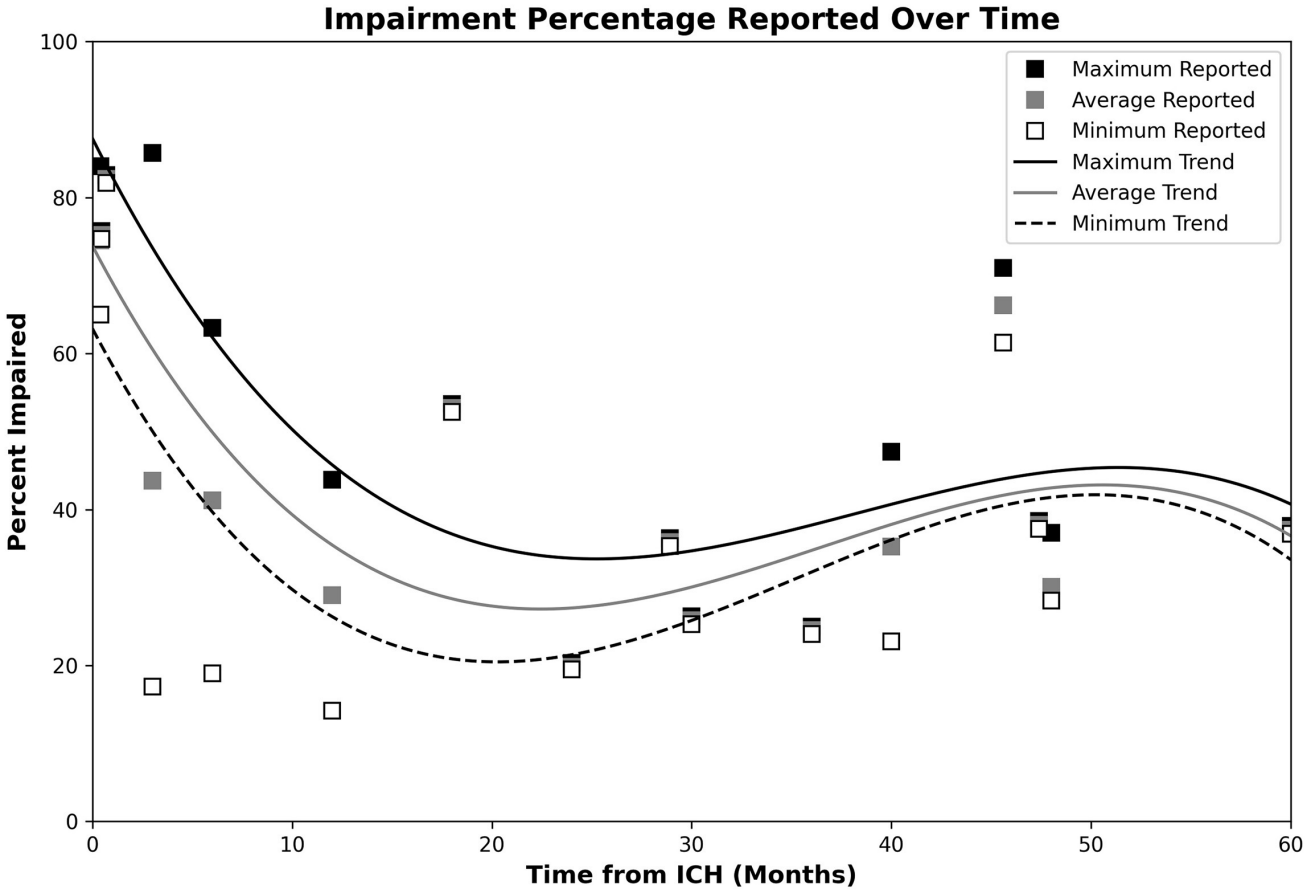
Plot of post-ICH CI percentages, with trendlines reflecting the maximum, minimum, and average observed CI proportions. Data for the maximum, average, and minimum data points are, respectively, shown by the black, gray, and open white markers. Respective CI trends are shown using black, gray, and dashed lines. Impairment appeared highest during the acute phase, dropped to a minimum during the 20–24-month timespan, and steadily increased over the remaining observed timespan.

Cognitive impairment reported after the onset of ICH varied considerably across the time horizon. Figure 2 depicts the proportions of ICH patients with post-ICH CI at various timepoints, with 3rd-order polynomial trendlines reflecting the maximum, minimum, and average observed CI proportions. Table 6 provides a summary of the significant imaging, demographic and clinical factors significantly associated with post-ICH CI across the studies included in this review.

**Table 6:** Summary of significant factors identified across reports included in the review.

**Imaging**			**Social**			**Clinical**		
**Factor**	**Significant**	**Reported**	**Factor**	**Significant**	**Reported**	**Factor**	**Significant**	**Reported**
Location	2	11	Age	8	18	Prior stroke/TIA	6	13
Hemorrhage volume	4	9	Sex/Gender	3	15	APOE E2	1	3
Laterality	2	9	Education	5	13	APOE E4	1	3
White matter hyperintensity severity	3	8	Ethnicity	2	3	Admission sBP	1	3
Intraventricular extension	1	7				C-Reactive protein	1	2
Microbleeds	4	6				White blood cell count	1	2
Atrophy	2	4				Mood disorder	1	1
White matter disease	1	1				Corpuscular volume	1	1
Cortical superficial siderosis (CSS)	3	3				Corpuscular hemoglobin	1	1
Multiple hemorrhages	1	2				Admission dBP	1	1
Leukoaraiosis	2	2				Mean sBP (24 h)	1	1
Dominant hemisphere ICH	1	1				NIH Stroke Scale	4	8
Cerebral small vessel disease score	1	1				Modified Rankin Scale	1	8
						Glasgow Coma Scale	1	4
						Pre-existing impairment	3	5
						Stroke during follow-up	1	1

The three acute phase studies reported that 84.0% of ICH patients demonstrated impairment in one or more cognitive domain at 12 days post-stroke (5, 16, 31), with 65.0% showing impairment in two or more domains (5), 75.2% of patients impaired at 2 weeks (16) and 82.4% of patients showing 2+ domain impairment at 3 weeks (31). Of the 3 studies, 1 assessed for delirium before testing (41) and 2 assessed for disorders of consciousness and aphasia (16, 31). Lobar and dominant-hemisphere ICH, and increased admission systolic blood pressure (SBP) were noted as factors associated with CI in this period (5, 16). Specifically, patients with lobar ICH showed higher rates of impairment in two or more domains (73.0% lobar vs. 58.0% non-lobar) while dominant-hemisphere hemorrhage and higher SBP showed respective odds ratios (OR) of 8.85 and 1.02 for impairment.

Impairment rates at 3 months post-ICH were reported to range from 17.3 to 40.2% (11, 15, 26, 37). Different studies demonstrated association between CI and Hispanic ethnicity (vs. non-Hispanic White) (18) and female sex (37)—Hispanic ICH patients presented with mental state examination (MSE) scores that were on the average 13.3 points lower than their non-Hispanic counterparts. Additionally, proportion of females with post-ICH CI during this time point was significantly higher than males (45.3 vs. 30.8%). Cognitive impairment during the 3 month post-ICH time period was also associated with increased hematoma volume—hazard ratio [(HR) 1.52 per 10 cc increase], and APOE ε2 genotype (HR: 1.69) (13), prior history of stroke, older age, higher baseline CI, and higher SBP during the first 24 h (37). Higher education was associated with lower rates of post-ICH CI (22), and separate reports associated 3-month post-ICH CI with both deep (25) and lobar ICH locations (13).

Studies reporting data from 6 to 12 months post-ICH found CI rates between 19.0 and 63.3% (12, 13, 16). Higher levels of CI were associated with increased age (HR: 1.05 per year), lower education level (HR: 1.78), pre-existing mild CI (HR: 5.85), and African American race (HR: 1.39) (12, 13, 28). Impairment was further linked to the severity of several cerebral small vessel disease (CSVD) markers such as, white matter hyperintensities per total brain volume (HR: 1.48), Fazekas score (HR: 1.51), number of cortical microbleeds (CMBs) ≥ 5 (HR: 2.05), white matter disease severity (HR: 1.70), and lobar CMB number (HR: 1.78) and APOE ε4 genotype (HR: 2.12) (12, 13). Visuospatial, executive, attention, and memory were observed as the primarily affected domains, and CI appeared to associated with the laterality of ICH (16, 33)—ICH in the left basal ganglia was linked to visuospatial impairment, while those with right basal ganglia ICH performed worse in linguistic tasks (33) and left sided ICH was associated with increased dementia development (38).

The proportion of ICH patients with CI 1 year post-ICH were reported at 14.2% (23) and 43.8% (19), with an estimated HR of 4.34 for the 3–12 months period (6) and incidences between 20.7 and 24.8 per 1,000 person-years (6, 40). One study reported that CI probabilities reduced and language performance improved between 3 and 18 months post-ICH, although the study was underpowered (7). Reports in the 2nd year post-ICH found CI rates of 19.8% at 2 years (23), while a second study reported 35.8% either became impaired or died at a median of 28.9 months (39). CI rates were reported between 19.1 and 24.5% (23, 24) at 3 years. The respective rates of CI and dementia were reported as 47.4 and 23.1% at 40 months, with the note that 65.4% of survivor showed some level of persistent deficit (38). CI rates that depended on the applied Montreal Cognitive Assessment (MoCA) threshold (61.4% at MoCA ≤ 23 and 71% at MoCA ≤ 25) (27) were reported at 3.8 years post-ICH, with a CI rate of 28.3% separately reported at 4 years post-ICH (23). Study at 5 years post-ICH reported incidence of 14.6 per 1,000 persons years, with a model-dependent HR of 2.14 and 2.17 (40). The only report of independent CI rates at this timepoint assessed initially asymptomatic ICH patients, who did not show any difference from healthy individuals (32). Long-term assessments estimated incidence rates of 11.8 per 1,000 individuals per year at 10 years (40) and 13.6 over the 1–10 year period (6). Long-term CI was found to be associated with the number of recurrent stroke episodes (36). Age, modified Rankin Scale (mRS) scores, and lobar location were found to be significant factors, with lower MoCA scores associated with higher age and greater disability (21, 27). Finally, impaired individuals were found to have higher baseline and discharge national institutes of health stroke scale (NIHSS) scores, with higher C-reactive protein levels (CRP) and white blood cell counts (WBC) (24).

### Cognitive Assessments

Across the included studies, the Information Questionnaire on Cognitive Decline in the Elderly (IQCODE) was utilized in 10 (31.3%) studies as the most-common test for pre-stroke cognitive function (11–13, 18, 19, 23, 25, 26, 29, 31) [including uses of the short-IQCODE (31), Spanish IQCODE (11), and French IQCODE (23)]. However, IQCODE cutoffs varied greatly across studies (Table 5). IQCODE assessments adopted 4 different thresholds for “cognitive decline” or “impairment” (IQCODE >3.5, >3.44, ≥ 3.4, 63 ≥ IQCODE ≥ 53) with 2 more thresholds (IQCODE ≥ 64, > 3.3) applied to describe dementia. No single threshold was adopted by more than two studies.

Whereas the IQCODE was used relatively consistently to assess pre-morbid cognitive status, the methodology of assessment for post-ICH CI was highly variable. Global cognition tests were commonly applied, with the Mini-Mental State Evaluation (MMSE) adopted by 14 (43.8%) studies (11, 12, 15, 19, 22, 23, 25, 26, 30, 32–34, 37, 38) and MoCA adopted by 5 (15.6%) (7, 16, 21, 27, 38). Thresholds set for CI included MMSE < 27, MMSE ≤ 24, MoCA < 26 and MoCA ≤ 23, with one study defining dementia at MMSE < 23 (30), and no threshold used by more than two studies (Table 5). Tiered CI definitions based on MMSE or MoCA were also established in three studies, with no studies sharing definitions (Table 5). One study adopted education-linked MMSE thresholds as a screening test for further assessment (22). Other studies made use of the Short MoCA (16), the Ascertain Dementia 8-item Informant (AD8) questionnaire (34), and the Modified MMSE (3MSE) (18), each with a singular use and threshold (short MoCA < 7, AD ≥ 2, 3MSE < 80) (Table 5). Studies based on telephone-based assessment adopted the modified Telephone Interview for Cognitive Status (TICS-m) (13), Telephone MoCA (T-MoCA) (7), or caregiver interview (39).

Beyond global cognition tests, 11 (34.4%) studies adopted test batteries to assess cognitive function, with the National Institute of Neurological Disease and Stroke—Canadian Stroke Network (NINDS-CSN) battery applied by two studies (7, 23) and the Frontal Assessment Battery (25), Consortium to Establish a Registry for Alzheimer's Disease (CERAD) battery (7), and Neuropsychiatric Battery (14) used by one study each. The remaining battery-based assessments used custom combinations of tests designed to assess different cognitive domains. In 13 (40.6%) studies, determination for CI was made based on general diagnosis (23, 36), DSM-IV (11, 14, 22, 24, 26, 29, 38), DSM-V (7), ICD-9 (40), and ICD-10 (6, 10, 19) diagnostic criteria. Finally, 5 (15.6%) defined CI by the relative decrease in function from baseline assessments (5, 28, 30, 33, 39).

### Imaging Methods and Assessments

Of the total 32 studies, 17 (53.1%) included imaging markers either in assessment or analysis [five computed tomography (CT) only, four magnetic resonance imaging (MRI) only, seven CT and MRI, one CT or MRI]. The most common imaging markers reported were ICH characteristics, including location (11), laterality (8) and volume (9). ICH location description varied between studies, with 7 different location definitions adopted (Table 5). ICH volume was commonly assessed (13, 21, 23, 25, 27, 28, 32, 37, 38), with intraventricular extension accounted for by 7 (12.9%) studies (13, 16, 21, 27, 33, 37, 38). Markers of CSVD and CAA were also assessed with regularity, although the individual markers were inconsistently reported. Major imaging features included the presence of CMBs (12, 13, 23, 25, 28), lacunes (12, 23, 28), White Matter Hyperintensity (WMH) volume (28), WMH Fazekas scoring (12, 13, 16, 23, 25, 27, 28, 30), presence of enlarged perivascular spaces (28, 32), and Cortical Superficial Siderosis (CSS) (23, 25, 28). CSS assessments were dichotomized according to presence/absence (25) or focal/disseminated (23, 28) status, with respective significance found for presence (25) and disseminated CSS (23, 28). One study extended imaging features to determine a total CSVD score, which was used both nominally and dichotomized (<3 or ≥3) (28). Finally, focal brain atrophy (32) and global cortical atrophy (12, 23, 33) were, respectively, assessed in one and three studies. Across studies, ICH volume, CMBs, atrophy, white matter disease, CSS, and leukoaraiosis showed the greatest levels of significance.

### Clinical and Sociodemographic Factors

The NIHSS was reported in 8 (25.8%) studies (7, 16, 18, 23–25, 29, 37). Functional outcomes were reported as the Barthel index in 3 (9.7%) (7, 29, 38), the mRS in 8 (25.0%) (7, 18, 21, 23, 25, 38, 39), and Activity of Daily Life measurement (38). Depression linked to ICH was assessed using the Beck Depression Inventory (21, 25), Montgomery–Asberg Depression Rating Scale (MADRS) (12, 25, 38), Geriatric Depression Scale (30), Hamilton Depression Rating Scale (22) and the Hospital Anxiety and Depression Scale (21). Stroke laterality was reported in two studies without imaging details (7, 29). Assessment on the Glasgow Coma Scale was reported in four studies (18, 27, 33, 37).

Among demographic factors, age and sex/gender were, respectively, accounted for in 18 (56.3%) (5, 12, 13, 16, 18, 21–25, 27–30, 32, 33, 37, 39) and 15 studies (46.9%, 10 sex, five gender) (12, 16, 18, 22–25, 27–30, 32, 37, 39, 41), with education assessed in 13 (40.6%) (5, 12, 13, 16, 18, 22, 23, 25, 27–30, 33). Notably, studies that did not address age and sex typically assessed ICH as a subgroup or as a case series. Pre-existing risk factors/comorbidities were most commonly reported for hypertension in 15 (46.9%) (13, 16, 18, 22, 24, 25, 27–30, 32, 35, 37–39), previous stroke or ICH in 11 (34.4%) (12, 13, 18, 22, 23, 25, 28, 32, 37–39), atrial fibrillation in 7 (21.9%) (12, 13, 18, 22, 23, 27, 30) and diabetes mellitus in 12 (37.5%) (13, 16, 18, 22, 24, 25, 27–30, 32, 37) studies. Anticoagulant therapy was not found to be a significant factor by either of the studies that assessed it (13, 37). The definition of education was inconsistent, and race/ethnicity was seldom reported, appearing in 3 (9.4%) studies and found to be a significantly associated factor by 2 (6.3%) (18, 32, 38). Marital status, assessed in one study (3.1%), was not significantly associated with post ICH CI (18). Two studies (6.3%) provided a greater emphasis on laboratory values and testing than other studies, including blood cell counts and protein levels (16, 24).

## Discussion

Our review identified 32 full-text studies that assessed post-ICH CI. With the exception of a single study with a small ICH sample size (*n* = 5) (30), all studies found CI to be relatively common after ICH with links to a variety of clinical, social, imaging, and genetic factors. Studies were largely comprised of cohort and case-control studies and provided information from a variety of different patient populations. Impairment rate information obtained from the reviewed texts suggests relatively high rates of CI during the acute phase of ICH that drop off over the first 2 years post-ICH and increase afterwards. Although rates of CI were consistently reported to be high during the acute post-ICH phase, these findings are likely be an over estimate due to factors directly and indirectly related to acute neurological injury such as, medications, infections/sepsis, and delirium. The alleviation of these transient factors, combined with the effects of true cognitive recovery, create an apparent decrease in CI rates over the following 20–22-month period. Long-term assessments then depict a secondary rise in CI rates, although these measurements demonstrate high variability and may be subject to survivorship biases. In studies with repeated testing, training effects did not appear to be a major concern based on long test-retest intervals and a common reliance on diagnostic criteria or patient-specific testing. This risk of CI and death was found to be greater after ICH than in other stroke types (40), with impairments appearing primarily within the domains of visuospatial processing, processing speed, nonverbal IQ, executive function, attention, and memory (5, 33). Patients presenting with severe aphasia during the acute phase were largely unstudied due to testing limitations, however the low number of exclusions across studies makes significant bias unlikely. There is modest evidence that ICH location influences the affected domains (25), and lobar/CAA ICH was associated with poorer recovery and greater rates of post-ICH CI (13, 16, 23, 27). Lobar ICH was also associated with increased age (41), however, and it is unclear if this relationship may mediate cognitive outcomes. Variability in patient CI percentage increased over time, with CI rates of 19.1 and 71.0% both observed in the 3–4 year timespan (23, 24, 27, 38).

Although hypertensive microangiopathy constitutes a primary ICH etiological mechanism (25, 42, 43) and acute post-ICH SBP was associated with early cognitive deficits (16), a clear relationship between chronic hypertension and the development of CI has yet to be established among ICH patients. Similarly, despite evident links between CI and both atrial fibrillation and diabetes mellitus in the general population and mixed stroke populations (29, 44–46), significant associations between these factors have not been reported in post-ICH cohorts. On the other hand, risk factors related to demographics and genetics (age, ethnicity/race, sex, and APOE genotype), presentation cerebral disease burden (CSVD characteristics, CSS, previous stroke, and hematoma volume) and other biological markers such as high WBC and CRP demonstrate a significant association with the development of post-ICH CI (12, 13, 16, 18, 21, 23, 24, 27, 28, 37).

Several of these factors—CSVD markers, CSS, and APOE genotypes—are also indicative of CAA, which has been associated to cognitive impairment and should be considered a condition of interest (17, 41, 47). The importance of acute-phase cerebral pathology is further supported by reports of similar CI rates observed between incidental asymptomatic ICH patients and their matched controls (32), and those with relatively preserved baseline cognitive status across the most commonly affected domains (visuospatial, executive, attention, orientation) (16, 41). Our review indicated that APOE ε2 and ε4 genotypes may, respectively, contribute to the early and late development of post-ICH CI (13). Contextually, this somewhat resembles the roles of the APOE genotypes in Alzheimer's disease (AD) and vascular dementia (VaD). Specifically, APOE ε2 has shown neuroprotective effects (48) and the early post-ICH deficits linked to it are transient, while APOE ε4 is a significant risk factor for VaD and AD (49) and the associated post-ICH CI show a delayed build over time. It is therefore possible that the role of APOE ε4 in the development of long-term CI among ICH survivors is related to pathways overlapping with VaD and AD, such as buildup of vascular risk factors, CAA, amyloid disease and aging, although this relationship will need to be clarified in future studies. Finally, increased attention should be paid to patient ethnicity/race, which as only addressed in 3 studies and found to be significant in 2. This may also suggest other factors of interest, as ethnicity often portends disparities social support, economic deprivation, healthcare access, and treatment (50–52). Present evidence suggests that post-ICH CI may result from combined effects of damage during the acute phase of hemorrhage and pre-existing underlying arteriopathy, however continued research into post-ICH CI will be necessary to explore both the clinical and societal factors that are critical for patient recovery during the post-ICH period.

The stated focus of study—assessing the development of CI after ICH—appears straightforward. Unfortunately, research in this direction is host to many inconsistencies within the applied methods and assessments. Across the 32 included studies, 20 different impairment and dementia thresholds based on global cognition tests, eight different ICH location classifications, and six different education thresholds were established. Consequently, cases exist where patients assessed with “dementia” by one study would not meet others' minimum classification for “cognitive impairment” (13, 29). These inconsistent definitions, combined with their seldom re-use, limit specific sub-analysis. Chosen assessments were presented across 16 different post-ICH timepoints, with 23 of the 32 studies testing at the 3, 6, and 12-month timepoints. Though we believe that this work provides a valuable summary of post-ICH CI, considerable variability in testing parameters adds significant uncertainty to the results and limits the ability to pool quantitative estimates across consistent timepoints. To assuage this, future efforts should seek to establish and adhere to a standardized assessment protocols, which can be supplemented with additional testing to suit specific research questions and domain areas. Efforts should also be made to specifically explore additional timepoints, particularly over an extended time horizon.

### Future Directions and Considerations

Although literature is beginning to emerge, several gaps persist in our understanding of post-ICH CI, its associated factors, and its temporal trajectories. Presently, the greatest needs appear to be those of standardization in measurement and assessment of CI along with precise quantification of pre-ICH cognitive function. This discussion is considered too large to be held within the context of this singular review, and a full consideration of existing assessment methods should be made within the context of the domains commonly affected by ICH. Larger cohorts of ICH patients with wide-ranging impairments and well-defined clinical, social, and radiological phenotypes also need to be assessed, using standardized scales and criteria at consistently defined and clinically meaningful time points over a long-term horizon of post-ICH recovery. The establishment of a concise and easily referenced testing methodology would also provide firm grounding for comparison and pooling of estimates across studies. Future studies are encouraged to, at minimum, account for attrition and survivorship biases, along with history, number, and recurrence of stroke-type events. Imaging studies should assess ICH volume, CMBs, and CSS at minimum, ideally assessing the full spectrum of CSVD markers and considering the potential for CAA. Assessments of potential race, ethnic, social, and sex disparities in post-ICH CI are also currently lacking, as is a clear depiction of how impaired domains may change over time. The design and implementation of future cohorts need to address these laminations such that disparate burden of post-ICH CI across population sub-groups can be studied and mitigated.

While the present review provides these suggestions, limitations within this study need to be acknowledged as well. First, the initial search mechanism was limited to the PubMed and ScienceDirect databases. Second, while data was assessed as thoroughly as possible some relevant information may have been lost through the exclusion of articles written in non-English languages. Finally, although efforts were made to investigate results, an effective meta-analysis could not be performed due to the variability in methods between papers. Future research should seek to expand on the present review through the inclusion of additional databases and non-English research, and it is our hope that increased standardization will allow for true meta-analysis to be performed in the future.

A two-stage systematic literature review was performed to comprehensively summarize current evidence on post-ICH CI. Evidence currently available in literature depicts a high rate of CI in the acute phase (<4 weeks), which drops to lower levels at 3 and 6 months. Rate of post-ICH CI then increases over time, although it seems to plateau at a level lower than that observed during the acute phase. Impairment onset generally appears to associate more with demographic and genetic variables and extent of ICH related cortical damage. The methods, timepoints, and markers used to assess CI are highly varied and limit the ability for quantitative evidence synthesis. This calls for consistent methods to be established for future investigations of post-ICH CI and dementia across heterogenous clinical phenotypes and population sub-groups.

## Data Availability Statement

The original contributions presented in the study are included in the article/supplementary material, further inquiries can be directed to the corresponding author/s.

## Author Contributions

TP performed data collection, extraction, and assessment, as well as manuscript writing. FV and TP designed the search strategy and focus. FV oversaw data collection, methods, and writing. FV, JM, and AP reviewed data quality. V-AL, MT, JM, AP, DW, SS, MS, and FV contributed to data interpretation and provided major revisions. All the authors contributed to manuscript revision and read and approved the submitted version.

## Conflict of Interest

The authors declare that the research was conducted in the absence of any commercial or financial relationships that could be construed as a potential conflict of interest.

## Publisher's Note

All claims expressed in this article are solely those of the authors and do not necessarily represent those of their affiliated organizations, or those of the publisher, the editors and the reviewers. Any product that may be evaluated in this article, or claim that may be made by its manufacturer, is not guaranteed or endorsed by the publisher.

## Abbreviations

CI: Cognitive impairment.
